# Increased Gray Matter Volume Induced by Chinese Language Acquisition in Adult Alphabetic Language Speakers

**DOI:** 10.3389/fpsyg.2022.824219

**Published:** 2022-04-25

**Authors:** Liu Tu, Fangyuan Zhou, Kei Omata, Wendi Li, Ruiwang Huang, Wei Gao, Zhenzhen Zhu, Yanyan Li, Chang Liu, Mengying Mao, Shuyu Zhang, Takashi Hanakawa

**Affiliations:** ^1^College of Foreign Studies, Jinan University, Guangzhou, China; ^2^Department of Advanced Neuroimaging, Integrative Brain Imaging Center, National Center of Neurology and Psychiatry, Kodaira, Japan; ^3^School of Psychology, Key Laboratory of Mental Health and Cognitive Science of Guangdong, Center for the Study of Applied Psychology and MRI Center, South China Normal University, Guangzhou, China; ^4^Center for Linguistics and Applied Linguistics, Guangdong University of Foreign Studies, Guangzhou, China; ^5^Higher Education Mega Center, Guangzhou, China; ^6^South China Business Trade College, Guangzhou, China; ^7^Integrated Neuroanatomy and Neuroimaging, Kyoto University Graduate School of Medicine, Kyoto, Japan

**Keywords:** VBM, gray matter, logographs, acquisition, plasticity

## Abstract

It is interesting to explore the effects of second language (L2) acquisition on anatomical change in brain at different stages for the neural structural adaptations are dynamic. Short-term Chinese training effects on brain anatomical structures in alphabetic language speakers have been already studied. However, little is known about the adaptations of the gray matter induced by acquiring Chinese language for a relatively long learning period in adult alphabetic language speakers. To explore this issue, we recruited 38 Indian overseas students in China as our subjects. The learned group included 17 participants who had learned Mandarin Chinese for an average of 3.24 years and achieved intermediate Chinese language proficiency. The control group included 21 subjects who had no knowledge about Chinese. None of the participants had any experience in learning logographic and tonal language before Chinese learning. We found that (1) the learned group had significantly greater gray matter volume (GMV) in the left lingual gyrus (LG) compared with the control group; (2) the Chinese characters’ reading accuracy was significantly and positively correlated to the GMV in the left LG and fusiform gyrus (FG) across the two groups; and (3) in the learned group, the duration of Chinese learning was significantly and positively correlated with the GMV in the left inferior frontal gyrus (IFG) after correction for multiple comparisons with small volume corrections. Our structural imaging findings are in line with the functional imaging studies reporting increased brain activation induced by Chinese acquisition in alphabetic language speakers. The regional gray matter changes reflected the additional requirements imposed by the more difficult processing of Chinese characters and tones. The present study also show that the biological bases of the adaptations induced by a relatively long period of Chinese learning were limited in the common areas for first and foreign language processing.

## Introduction

Research has convincingly demonstrated that there are both common and distinct neural mechanisms across alphabetic languages and Chinese. Orthographic depth is the transparency of spelling-to-sound mapping of the words or characters in a language. Compared with alphabetic words, Chinese characters are orthographically deeper, for Hanzi consists of graphic components called radicals arranged in different ways such as side by side, top to bottom, or inside-outside, while alphabetic words have linear combinations of letters. Resultantly, Chinese writings are visually more complicated than alphabetic writings. Reading both alphabetic languages and Chinese words recruits the left lingual gyrus (LG), left fusiform gyrus (FG), and inferior occipital gyrus (IOG) for words visual processing (Nelson et al., 2009). However, alphabetic languages show strong left-lateralized effects in visual areas while Chinese shows bilateral effects in visual areas, as reported in several meta-analysis (Bolger et al., 2005; Tan et al., 2005; Zhu et al., 2014; Martin et al., 2015; Zhang et al., 2018). Unlike non-tonal languages, every syllable of Chinese carries a tone. Syllables composed of identical segments but carrying different tones differ in meaning. Tonal and non-tonal languages show large cross-linguistic commonalities in the neural processing (Chien et al., 2020), but compared with non-tonal languages processing, Chinese processing induces increased activity in the bilateral fronto-parietal regions (Li et al., 2010), fronto-temporal areas (Kwok et al., 2016), or bilateral temporo-parietal semantic regions and subcortical areas (Chien et al., 2020) in native Chinese speakers.

Chinese (L2) learning-induced increases in brain activation for alphabetic language speakers have been already found in the regions including the bilateral LG, left FG, left inferior parietal lobule, and left IFG (Deng et al., 2011; Zhao et al., 2012; Cao et al., 2017; Tian et al., 2019). Deng et al. (2011) studied the neural changes related to the learning of the pronunciation of Chinese characters in English speakers by using fMRI. They found the training-related effects in several regions including the bilateral LG and left IFG, and demonstrated that better learning was correlated with increasing activation in the left IFG. Zhao et al. (2012) examined the neural basis of phonological processing in Chinese later acquired as an L2 and reported that the L2 learners exhibited greater activation in the ventral aspects of the left inferior parietal lobule and left IFG for irregular character reading minus regular characters reading. Cao et al. (2017) reported that Chinese words elicited greater activation in the left IFG, left inferior temporal gyrus and left FG than Spanish words, while no region was more activated in Spanish than in Chinese in native English-speaking adults who learned Chinese and Spanish over a 2-weeks period. Tian et al. (2019) examined the phonological consistency effect of Chinese characters in non-native speakers who studied Chinese as an L2 for at least 1 year and found that the effect was mainly in the left LG, left FG, and left precentral gyrus extending to the left IFG.

Since the structure-function correspondences have been revealed by recent studies (Wong et al., 2007, 2008; Luk et al., 2011; Abutalebi et al., 2012; Gold et al., 2013; Hosoda et al., 2013), indicating that anatomical changes are the foundation of the functional engagement and modulation, it is therefore interesting to investigate the effect of Chinese (L2) acquisition on brain structures to know whether and how the recruitment of common and distinct neural mechanisms across alphabetic and Chinese language affects the anatomical change in the brain.

Indeed, there are a few studies up to date demonstrating that learning Chinese changes the gray matter density, cortex thickness and white matter connectivity in the language processing and language control networks (Crinion et al., 2009; Schlegel et al., 2012; Qi et al., 2015; Legault et al., 2019). Among them, Crinion et al. (2009) compared structural brain images in three groups of participants: 31 native Chinese speakers; seven native English speakers who had learned Chinese in adulthood; and 21 European multilinguals who did not speak Chinese. The results identified two brain regions in the vicinity of the right anterior temporal lobe and the left insula where speakers of Chinese had significantly greater gray and white matter density compared with those who did not speak Chinese. However, the authors pointed out that the “sample size of multilingual non-native Chinese-speaking group was small, the fact that we observed structural differences in this group is likely to be a consequence of homogeneity in their proficiency, how they learned Chinese and their daily use of Chinese.” In addition, several studies focused on short-term intensive Chinese training, such as 20 days of training in the study of Legault et al. (2019); a 9-month spoken and written Modern Standard Chinese course in the research of Schlegel et al. (2012) and a 4-weeks intensive Modern Standard Mandarin training in the study of Qi et al. (2015). Legault et al. (2019) investigated changes in cortical thickness and GMV in response to short-term Chinese (L2) vocabulary learning. The L2 training groups learned the same 90 Mandarin Chinese nouns across seven training sessions over 20 days. The results showed cortex thickness and GMV increased in regions implicated in a language control network for both L2 training groups. In addition, Schlegel et al. (2012) reported brain structural plasticity elicited by Chinese learning for adults in white matter tracts was associated with traditional left hemisphere language areas and their right hemisphere, and the most significant changes occurred in frontal lobe tracts crossing the genu of the corpus callosum. Qi et al. (2015) demonstrated that greater initial fractional anisotropy (FA) in both the right superior longitudinal fasciculus (parietal bundle) and right inferior longitudinal fasciculus was associated with more successful Mandarin learning.

It is important to note that the structural plasticity in the bilingual brain induced by L2 acquisition is dynamic and depends on the quantity and quality of the language learning and switching experience (Pliatsikas, 2020). Therefore, it is valuable to explore the effect on anatomical change induced by short-term training as well as different stages of language acquisition in which there may be varied increases or decreases in gray and white matter integrity during the language acquisition. However, little is known about whether a non-alphabetic language learning for a relatively long period, for example, 2 or 3 years, might induce structural neuroplasticity in natively alphabetic language speakers.

To explore the issue, we recruited subjects who had had no experience of learning any logographic or tonal languages before Chinese learning as the experimental group and those who had not studied Chinese or any other logographic or tonal languages as the control group. All of the subjects in the experimental group acquired Chinese in an immersed environment (in China) for a relatively longer time (3.24 years on average) and achieved intermediate proficiency in Chinese mandarin. Recent structural MRI studies have showed that neuroplasticity as a function of second language learning is dependent on several key variables, namely bilingual experience, cognitive control experience, age of acquisition, and proficiency in the L2 (Li et al., 2014). In this study, we tried to control the potential confounding factors which may affect the anatomical structure of the subjects. First, AoA of L2. Studies have reported that AoA of L2 plays an influential role in modulating brain function and structure for acquiring languages (Mechelli et al., 2004; Klein et al., 2014; Wei et al., 2015; Liu et al., 2017, 2020, 2021; Tu et al., 2020). In this study, most of the subjects were simultaneous bilinguals and all the participants’ AoA of L2 in this study was less than 6 years old, which is believed to be an important cutoff age when considering the AoA effect on brain functional and structural plasticity. Second, musical training experience. Musical training influences neural bases of language processing (Schön et al., 2004; Moreno et al., 2009). Even for genetically identical twins, there were within-pair differences in the auditory-motor network of the left hemisphere and more developed white matter microstructure in relevant tracts in both hemispheres and the corpus callosum between the playing and non-playing siblings (de Manzano and Ullén, 2018). We recruited the subjects without any experience in musical training. Third, the experience of acquiring languages. The number of languages acquired by the subjects in the learned group ranged from 2 to 6 before Chinese learning, which meant that mandarin Chinese was at least their third language, avoiding the possible explanation that learning any new language instead of learning Chinese would have an effect on the anatomical plasticity. Finally, we did a Raven test for the participants.

Dual/multiple language processing relies on the language processing system as well as the cognitive control system. All the participants in this study were early bilinguals/multilinguals (all of the 38 subjects acquired their second language before 6 years old, and among them, 27 of the subjects were simultaneous bilinguals who acquired the first and second language before 3 years old), who already had rich experience of learning new languages, meaning that their cognitive control system and language processing system had already been well established since they were young. What was new to them was the learning of the logographic and tonal language. Therefore, we did not expect structural plasticity in the language control network but in the logographic and tonal processing network in the learned group compared with the control group.

Based on the previous functional studies about Chinese learning-induced increases in brain activation for alphabetic language speakers who had been learning Chinese as L2 (Deng et al., 2011; Zhao et al., 2012; Cao et al., 2017; Tian et al., 2019), we expected the structural change in the bilateral LG, left FG, left inferior parietal lobule and left IFG, and we also expected the correlation between the brain changes in these areas and the individual differences, especially, of the duration of Chinese learning, and of the accuracy and reaction time in Chinese characters recognition tasks. We were also interested in knowing how the effect would happen in the language-general and/or language-distinct areas across languages.

## Participants

All the 38 participants were Indian overseas students in universities in China (Table 1). For the learned group, we recruited 17 participants (11 males/6 females, 21.00 ± 1.17 years old) who had learned mandarin Chinese for 3.24 years on average (range 1--10 years; SD 1.99 years), having achieved intermediate Chinese proficiency and passed the third level of HSK (Hanyu Shuipin Kaoshi).^1^ HSK is an official, standardized exam used by the China National Office for Teaching Chinese as a Foreign Language to test learners’ mastery of Mandarin as a foreign language. According to the official statement, those who reach HSK Level 3 can complete basic communication tasks in daily life, study, and work. If traveling in China, Level 3 can handle most of the communication tasks they encounter. Their vocabulary in Chinese is about 600–1,200. Their proficiency equals B1–B2 in the Common European Framework Reference for Languages (CEFR). For the control group, we recruited 21 Indian overseas students (10 males/11 females, 18.10 ± 1.10 years old) without any experience of Chinese learning who had arrived in China a month before the scanning.

**TABLE 1:** Demographic characters and behavioral performance of all subjects.

	Learned group	Control group	Statistics	*p*-value
	*n* = 17	*n* = 21		
**Sociodemographic features**				
Gender (male/female)	11/6	10/11	χ*^2^* = 1.1	0.29
Age (years old)	21 ± 1.17	18.10 ± 1.09	*t* = –7.89	<0.0001
Raven’s performance	59 ± 23.92	47.67 ± 27.30	*t* = –1.34	0.19
Age of acquisition (L2)	2.53 ± 1.88	2.81 ± 2.16	*t* = –0.42	0.68
**Chinese characters recognition**				
Accuracy	0.73 ± 0.09	0.58 ± 0.15	*t* = –3.8	0.01
Reaction time	434.3 ± 140.7	428.6 ± 142.6	*t* = –1.2	0.903
**HSK score**				
Oral	72.41 ± 9.00	N.A.	N.A.	N.A.
Writing	78.77 ± 11.37	N.A.	N.A.	N.A.
Total score	151.2 ± 17.95	N.A.	N.A.	N.A.

Across all the participants, their AoA of L2 was less than 6 years old. The numbers of the languages they could speak ranged from 2 to 6, and the languages they had acquired included English, Hindi, Khasi, Bengali, Behari, Punjalu, Himachali, Tamil, Arabi, Gujarati, Kannada, Telugu, Malayalam, Urdu, Bengali, Malayalam, Oriya, Haryanvi, and Mandarin Chinese. All the languages they acquired except Mandarin Chinese are alphabetic languages. For example, Hindi and Urdu languages belong to the Indo-Aryan language family. Hindi uses the Devanagari writing system which has both syllabic and alphabetic properties while Urdu is written in Nasta’liq, a form of Arabic script. All participants were right-handed, as assessed by performance on a Handedness Questionnaire (Snyder and Harries, 1993). Participants did not have any untreated visual or auditory deficits or any history of neurological disorders.

## Behavioral Tasks

**FIGURE 1:**
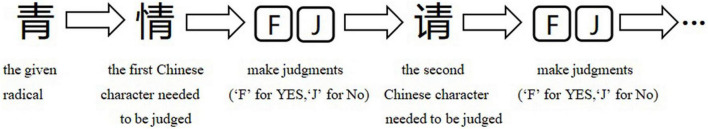
Process of Chinese Hanzi recognition task. The participants were requested to make judgements on whether the presented Chinese Hanzi (10 characters in one group) included the given radical in each group. The stimuli were presented for 2.5 s followed by the appearance of a fixation cross for the other 2.5 s. For example, the given radical “青” was presented to the participants first, and then “情” was presented. The participants were required to make judgements on whether the Chinese character “情” included the radical “青”. Since “情” included “青” here, if the participants pressed “F” to show “yes” here, they made a correct judgment. Then the next character “请” was presented, the participants were required to judge again on whether “请” included “青”. 6 groups were tested in total. In each group, one radical and 10 Chinese characters were given to make judgements. All of the stimuli are listed in Table 2.

In the Chinese Hanzi recognition task, the participants were requested to make judgements on whether the presented Chinese Hanzi included the given Chinese radicals (totally 6 groups of 10 Chinese Hanzi were given) (Figure 1 and Table 2). The participants were requested to make judgements on whether the presented Chinese Hanzi (10 characters in one group) included the given radical in each group. The stimuli were presented for 2.5s followed by the appearance of a fixation cross for the other 2.5s. For example, the given radical “青” was presented to the participants first, and then “情” was presented. The participants were required to make judgements on whether the Chinese character“情” included the radical “青”. Since “情” included “青” here, if the participants pressed “F” to show “yes” here, they made a correct judgement. Then the next character “请” was presented, the participants were required to judge again on whether “请” included “青”. Six groups were tested in total. In each group, one radical and 10 Chinese characters were given to make judgements. All of the stimuli are listed in Table 2.

**TABLE 2:** Stimuli in the Chinese characters recognition task.

The given radicals	Chinese characters needed to be judged
青	情、请、取、清、箐、散、氰、精、静、靓
田	男、画、亩、甲、畜、略、查、由、电、甸
木	林、李、术、困、拦、休、体、杭、种、栏
土	培、仕、共、基、垣、域、试、坛、壮、坦
走	赶、匙、迁、趋、赴、通、趟、题、赵、超
斤	斧、欣、牌、斩、斥、牍、新、列、牊、锦

## Image Acquisition

Image data was obtained on a 3-T Siemens Magnetom Trio MRI scanner with a 12-channel head coil in the Brain Imaging Center at the South China Normal University. We acquired 3-dimensional T1-weighted images using the Magnetization Prepared Rapid Gradient Echo (MP-RAGE) sequence with following parameters: repetition time (TR) = 2,300 ms, echo time (TE) = 3.24 ms, inversion time (TI) = 900 ms, flip angle = 9°, field of view = 256 mm^2^, and voxel size = 1 × 1 × 1 mm^3^, and 172 sagittal slices.

## Data Analysis

For data analysis, we used Voxel-Based Morphometry(VBM) approach, which is the most commonly used method for the study of gray matter (Good et al., 2001). The structural brain images were preprocessed using Statistical Parametric Mapping software (SPM12; Wellcome Trust Center of Imaging Neuroscience)^2^ running under Matlab R2019b (MathWorks, Natick, MA, United States).

To ensure a successful initial spatial registration, each MRI image was visually checked for artifacts and gross anatomical abnormalities and manually reoriented to the origin (anterior commissar–posterior commissure line) primarily. Then these reoriented images were segmented into gray matter, white matter, cerebrospinal fluid (CSF), and other three background classes. Next, the segmented images were input into a high-dimensional template created by DARTEL(diffeomorphic anatomical registration through exponentiated lie algebra) tools to increase the accuracy of inter-subject registration, and then these images were further transformed into the Montreal Neurological Institute (MNI). Finally, image data were spatially smoothed with an isotropic Gaussian kernel of 8 mm FWHM (full-width half-maximum).

Regions with significant differences between the learned group versus the control group or correlation with behavioral parameters were identified using a statistical threshold of *P* < 0.05 following correction for multiple comparisons either across the whole brain or within predetermined regions of interest (ROIs) for small volume correction analysis. Studies performing VBM used small volume corrections as a way of limiting the analysis to specific regions without suffering from the problems of ROIs averaging (Garcia-Penton et al., 2016). Some studies of neuroanatomy of bilingualism performed small volume corrections in their VBM analysis (Grogan et al., 2012; Ressel et al., 2012; Zou et al., 2012; Abutalebi et al., 2013; Pigdon et al., 2019).

We failed to find significant differences between the two groups correcting for multiple comparisons across the whole brain. We used the small volume correction in the test for the differences in GMV between the learned group and the control group. Sex, ages, AoA-L2, the number of languages acquired, and Raven’s performance of the participants were considered as covariates and then were regressed out in the statistical analysis. Across both groups and in the learned group and control group respectively, we tested for the regions where GMV was correlated with Chinese characters’ reading accuracy and reading reaction time; in the learned group, we tested for the regions where GMV was correlated with the Chinese learning in years while the effects of sex, ages, AoA-L2, the number of languages learned, and the Raven’s performance of the participants were removed.

For the small volume correction analysis, we used ROIs (15-mm radius) which centered at the co-ordinates in the bilateral lingual gyri (x = –27, y = –93, z = –12; x = 15, y = –78, z = –18), left FG(x = –45, y = –63, z = –16), left inferior parietal lobule(x = –49, y = –49, z = 21), and left IFG (x = –48, y = 6, z = 32) (Deng et al., 2011; Zhao et al., 2012; Cao et al., 2017; Tian et al., 2019). For this study, we transformed the previous Talairach coordinates into MNI coordinates using a Matlab script from https://bioimagesuiteweb.github.io/webapp/mni2tal.html. We reported significant differences between the groups using small volume corrections family-wise error rate (FWE) corrected (*P* < 0.05) for the voxels within the anatomical ROIs.

## Results

### Behavioral Results

In the Hanzi recognition task, the accuracy of the learned group was significantly higher than that of the control group (*p* = 0.01); the two groups did not show any difference in their reaction time in the Chinese characters recognition task. We tested the relationship between the accuracy of Chinese Hanzi recognition and the HSK score of the learned group by performing a Pearson correlation analysis. The result revealed a positive correlation between the accuracy of Chinese Hanzi recognition and the HSK score (*p* = 0.003), supporting that the accuracy of Chinese Hanzi recognition reflected their Chinese proficiency level well.

### Imaging Results

**FIGURE 2:**
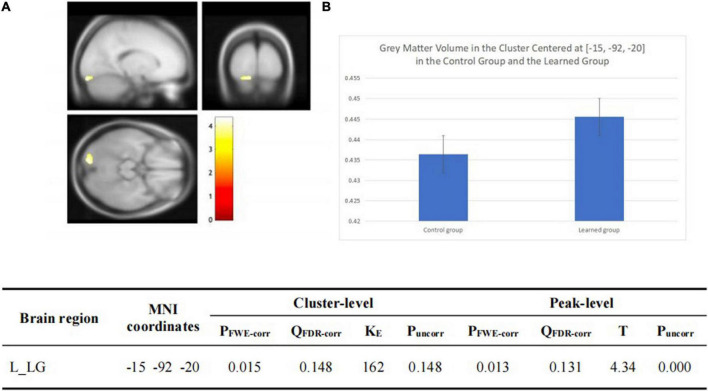
**(A)** Statistical parametrical maps (threshold at *P* < 0.001, uncorrected for display purposes) showing regions of increased gray matter volume in the Chinese learned group when compared with the control group via VBM. Sex, ages, AoA-L2, the number of languages learned, Raven’s performance were considered as covariates and then were regressed out in the statistical analysis. Region in the left LG showed significant difference after correction for multiple comparisons using small volume corrections (*P* < 0.05). **(B)** Greater GMV in the cluster centered at (–15, –92, –20) in the learned group than the control group.

**FIGURE 3:**
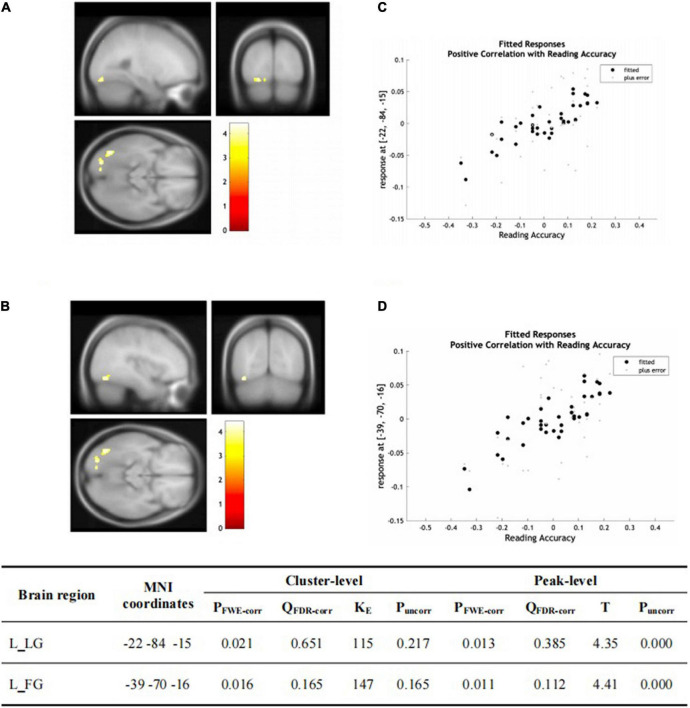
**(A,B)** Statistical parametric maps (threshold at *P* < 0.001, uncorrected for display purpose showing the results from the regression analysis of Chinese characters’ reading accuracy on grey matter volume using VBM). Sex, ages, AoA-L2, the number of languages learned, Raven’s performance were considered as the covariates and then were regressed out in the statistical analysis. Increased GMV in left LG [showed in **(A)**] and left FG [showed in **(B)**] was associated with higher accuracy of Chinese characters recognition for all the subjects, respectively. This result was significant after correction for multiple comparisons using small volume corrections (*P* < 0.05). **(C)** GMV in the cluster centered at (–22, –84, –15) correlates with reading accuracy of Chinese characters across the two groups in the left LG. **(D)** GMV in the cluster centered at (–39, –70, –16) correlates with reading accuracy of Chinese characters across the two groups in the left FG.

**FIGURE 4:**
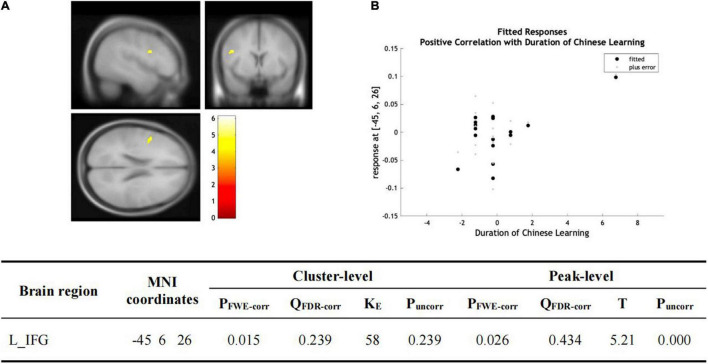
**(A)** Statistical parametric maps (threshold at *P* < 0.001, uncorrected for display purpose showing the results from the regression analysis of Chinese learning time on gray matter volume using VBM). Sex, ages, AoA-L2, the number of languages learned, Raven’s performance were considered as the covariates and then were regressed out in the statistical analysis. Increased gray matter volume in left IFG was associated with longer Chinese learning time for the subjects who had learned Chinese. This result was significant after correction for multiple comparisons using small volume corrections (*P* < 0.05). **(B)** GMV in the cluster centered at (–45, –6, –26) correlates with the reading accuracy of Chinese characters in the left IFG.

There was no significant group-wise difference in GMV in the whole-brain analysis. However, we found (1) significantly greater GMV in the learned group than the control group in the left LG (x = –15, y = –92, z = –20; *P* = 0.015); (2) the Chinese characters reading accuracy was positively correlated to GMV in the left LG (x = –22, y = –84, z = –15; *p* = 0.021) and left FG (x = –39, y = –70, z = –16; *p* = 0.016) across the two groups; and (3) the duration of Chinese learning was positively correlated to GMV in the left IFG(x = –45, y = 6, z = 26; *p* = 0.015) in the learned group after correction for multiple comparisons with a small volume correction analysis in a spherical volume of 15 mm radius which centered on the coordinates of the ROIs we have chosen (Figures 2–4).

## Discussion

We found significantly greater GMV in the learned group than in the control group in the left LG; the positive correlation between the Chinese characters’ reading accuracy and the GMV in the left LG and the left FG across the two groups; and the positive correlation between the Chinese learning time and the GMV in the left IFG. The left LG, FG, and IFG, all three areas are involved in language processing. The left LG played a crucial role in the integration of language independent semantic information across production and word reading (Van de Putte et al., 2018) and showed evidence of selectivity for sub-lexical phonological and semantic cues (Yang et al., 2011). The left FG has been reported to have an important role in long-term representations of Chinese orthographic lexicon and be modulated by character frequency (Chen et al., 2016; Yang et al., 2018), and showed bottom-up orthographic effects on semantic representations (Fan et al., 2010). It has been suggested that the pars opercularis of the left IFG was involved in grapheme-to-phoneme conversion (Fiebach et al., 2002).

Of note, our findings are consistent with functional and structural imaging studies of language processing recruitment. Functionally, the coordinates (–15, –92, –20; –22, –84, –15) of the cluster in the LG and the coordinates (–39, –70, –11) of the cluster in the FG we identified in this study are close to the coordinates (–23, –95, –18; –37, –76, –21) in Chinese characters reading (Bolger et al., 2005) and (–44, –74, –4) in English/French/German words reading (Martin et al., 2015) in their meta-analysis studies, additionally, the coordinates (–18, –92, –4; –40, –78, –10) in a conjunction analysis in Chinese and English reading for the subjects who were 1st grade monolingual and monoliterate children in Chinese and English (Krafnick et al., 2016).

Our structural imaging findings are in line with functional imaging studies reporting increased brain activation induced by Chinese acquisition for alphabetic language speakers (Deng et al., 2011; Zhao et al., 2012; Cao et al., 2017; Tian et al., 2019). For example, Chinese words elicited greater activation in the left IFG and FG as compared to Spanish words for native English-speaking adults who learned Chinese words as well as Spanish words (Cao et al., 2017). When the Chinese characters presented were more difficult to process, e.g., inconsistent words compared with consistent words in Tian et al. (2019)’s study; irregular characters versus regular characters in Zhao et al. (2012)’s study, more neural resources in the areas such as the left LG, FG, and IFG were required.

The increased GMV found in the LG in this study may demonstrate the neural adaptations induced by Chinese learning, for the languages our subjects had acquired before Chinese acquisition are all alphabetic languages. We propose that acquiring Chinese language increased the demand on the neural resources required for processing orthographically deeper Chinese characters and syllables composed of identical segments but carrying different tones which differ in meaning. The regional gray matter changes reflected the additional requirements imposed by the more difficult processing of directly retrieving stored phonological representations for the whole words and processing tonal pitch as semantic and phonological information in Chinese.

It’s very interesting to have found the significant and positive correlation between Chinese characters’ reading accuracy and GMV in the left FG and LG across the two groups, and between Chinese learning time in years and GMV in the left IFG in the learned group. These results are consistent with the previous functional and structural studies. For instance, functional activation of the FG was correlated with better word reading for beginning readers aged 5–6 years (Centanni et al., 2018). Structurally, in a study with a large sample of 226 native Chinese subjects, Zhang et al. (2013) reported the cortical thickness in the left middle FG was positively correlated with performance on reading Chinese characters in the coordinates (–38, –63, –17) and English words in the coordinates (–39, –66, –17), which are also close to the coordinates of our results. In addition, more successful learners showed greater IFG activation in functional networks (Turkeltaub et al., 2003; Jones et al., 2012; Yang et al., 2015); and better Chinese learning was correlated with increasing activation in the left IFG (Deng et al., 2011). As to the learning time effect, a longitudinal fMRI study (Kuper et al., 2021) tracked functional changes in the reading network of beginning learners of Greek over 1 year and found the interaction between language and learning time in a semantic decision task involved brain regions including the left IFG whose peak coordinates (–42, 6, 28) were close to ours (–45, 6, 26), and FG whose peak coordinates (–48, –60, –10) laid close to ours (–39, –70, –16). In our study, GMV in visual areas across the two groups was sensitive to the Chinese characters’ reading accuracy instead of Chinese learning time while GMV in the left IFG in the learned group was sensitive to Chinese learning time instead of other individual differences in behavior. It seemed that the changes of GMV in the left LG and FG were independent of, but the change of GMV in the left IFG depended on Chinese learning time.

It is important to note that the acquisition of Chinese as a foreign language for our participants was accompanied by structural adaptations in the brain regions including the left LG, FG, and IFG, which are common instead of distinct regions for alphabetic as well as logographic language processing (Bitan et al., 2005; Xue et al., 2006). The left LG, FG, and IFG are key areas involved in visual word recognition in alphabetic languages as well as in Chinese (Liu et al., 2003; Bolger et al., 2005; Wu et al., 2012; Krafnick et al., 2016). A meta-analytic study of the neural systems for auditory processing of lexical tones showed that the largest cluster with the highest convergence was located in the left IFG including BA44 for auditory processing of lexical tones in tonal languages as well as in non-tonal languages (Kwok et al., 2017). Phonological learning of novel visual words induced increases in the left IFG and FG for alphabetic as well as non-alphabetic scripts (Bitan et al., 2005; Xue et al., 2006). Thus, our results indicated that for the experienced alphabetic language multilingual Indians in our study, the biological bases of the adaptations induced by a relatively long period of Chinese learning were limited in the common areas for first and foreign language processing.

Alphabetic languages have showed strong left-lateralized effects in visual areas while Chinese have showed bilateral effects in visual areas, as reported in several meta-analyses (Turkeltaub et al., 2002; Bolger et al., 2005; Tan et al., 2005; Zhu et al., 2014; Martin et al., 2015). In addition, white matter change in the right hemisphere was found in the short-term Chinese (L2) training (Schlegel et al., 2012; Qi et al., 2015). However, we have not found any effect in the right hemisphere in our subjects in this study. One of the possible reasons is that there might be some structural effects in the right occipital gyrus but it was not large enough to be detected because of the small sample of the subjects and the intermediate level of their Chinese proficiency. After all, our subjects have only reached the intermediate Chinese proficiency, only knowing about 600–1,200 Chinese characters. The neural demand required for logographic words’ visual processing for them, for example, in fast and difficult content reading, was not high. If they had reached a higher Chinese language level, they might have a right hemisphere anatomical change to support the processing of the more reading demands.

Another possible reason is the influence of the existing neural correlates on language processing for the subjects. It was reported that the existing neural resources for L1 processing were also used in L2 processing. For example, L2 activation was similar to the L1 activation when Korean-English and Chinese-English speakers performed a visual rhyming judgement task and were compared to L1 control groups (Kim et al., 2017). Interestingly, at the beginning of a new characters-learning, for adult native Chinese speakers, who were expected to process characters in the fusiform bilaterally, both alphabetic and logographic artificial language reading training only induced significant increased neural activities in the left fusiform, but not in the right fusiform (Mei et al., 2013). We speculate that in Mei et al. (2013)’s study, the established ability to process relatively sophisticated Chinese characters made the subjects easily recognize the comparatively simpler artificial words, making them only use the left fusiform to process the artificial words. In addition, Manuel et al. (2009) reported learning to read Spain, an alphabetic language, in illiterate adulthood had a significant effect on the structure of bilateral dorsal occipital gyri and middle temporal gyri although Spain is an alphabetic language. All the subjects were adult illiterates without any experience in characters processing before learning Spain. They had not established any neural circuit to process visual words before learning Spain. It seems that whether the right hemisphere is involved depends on the more or less demanding of visual processing of the words or characters. Interestingly, all of our subjects reported that it was easy for them to learn a foreign language, including Chinese because they had mastered at least two languages, some of them 3–5 languages before Chinese learning. Therefore, we propose that it is possible that the Indians only used their well-established left-lateralized language areas to process Chinese, which eventually changed the GMV of the common regions for alphabetic as well as logographic language processing.

The age difference between the learned group (21 ± 1.17 years old) and the control group (18.10 ± 1.09 years old) might induce a cofounding effect. However, we factored out the age of the two groups as the covariant when we did the image data analysis to regress out the effect of age. In addition, accumulating MRI longitudinal studies reported a linearly decrease in total GMV (Lebel and Beaulieu, 2011; Aubert-Broche et al., 2013; Tamnes et al., 2013; Wierenga et al., 2014) and regional gray matter in temporal and occipital gyrus (Aubert-Broche et al., 2013) between late childhood and the early twenties. Instead of the expected decreased GMV based on the studies, we found the increased GMV here.

## Limitations

Firstly, the subjects only reached the intermediate level of Chinese, limiting the explanation of the results we found here, so a longitudinal study is expected in the future. Secondly, this research focused only on the effect on the participants who did not have any experience on logographic or tonal language learning. Other research exploring the Chinese-characters effect on those who have experience of logographic language, for instance, Japanese kanji, will reveal more about the Chinese (L2) acquisition effect.

## Conclusion

We compared the structural differences in brain between Indian overseas students who stayed in China for 2–3 years and achieved intermediate Chinese proficiency with those students who had arrived in China a month before and had yet to study Chinese. Having controlled their age, AoA-L2, raven scores, musical training experience and the number of languages acquired, we found (1) significantly greater GMV in the learned group than the control group in the left LG; (2) the significant positive correlation between Chinese characters reading accuracy and GMV in the left LG and FG across the two groups; and (3) the significant positive correlation between Chinese learning time and GMV in the left IFG. The regional gray matter changes reflected the additional requirements imposed by the more difficult grapheme-to-phoneme and phonology-to-meaning conversion in Chinese words processing. Notably, the GMV in the left LG and FG predicted the Chinese characters recognition and the GMV in the left IFG was sensitive to Chinese language learning time. In addition, all the regions we identified here were related to common instead of distinct neural mechanisms across alphabetic languages and Chinese language, indicating that for the experienced alphabetic language multilinguals, the biological bases of the adaptations induced by a relatively long period of Chinese learning were limited in the common areas for first and foreign language processing. To the best of our knowledge, this is the first study exploring the effect on anatomical plasticity in brain induced by the Chinese acquisition in an immersed environment for a relatively long learning period in adult alphabetic language speakers.

## Data Availability Statement

The raw data supporting the conclusions of this article will be made available by the authors, without undue reservation.

## Ethics Statement

The studies involving human participants were reviewed and approved by the Medical Ethics Committee of Jinan University. The patients/participants provided their written informed consent to participate in this study. Written informed consent was obtained from the individual(s) and minor(s)’ legal guardian/next of kin, for the publication of any potentially identifiable images or data included in this article.

## Author Contributions

LT and TH contributed to conception and design of the study. LT, RH, WG, ZZ, YL, and CL performed the experiment. LT and KO performed the statistical analysis. LT wrote the first draft of the manuscript. FZ, WL, MM, SZ, and TH wrote sections of the manuscript. All authors contributed to manuscript revision, read, and approved the submitted version.

## Conflict of Interest

The authors declare that the research was conducted in the absence of any commercial or financial relationships that could be construed as a potential conflict of interest.

## Publisher’s Note

All claims expressed in this article are solely those of the authors and do not necessarily represent those of their affiliated organizations, or those of the publisher, the editors and the reviewers. Any product that may be evaluated in this article, or claim that may be made by its manufacturer, is not guaranteed or endorsed by the publisher.

## Footnotes

1
www.chinesetest.cn

2
https://www.fil.ion.ucl.ac.uk/spm
